# Retrospective analyses of heartworm (*Dirofilaria immitis*) disease and ectoparasite preventive medication compliance in veterinary practices in the USA

**DOI:** 10.1186/s13071-023-05735-y

**Published:** 2023-04-28

**Authors:** Kennedy Mwacalimba, Derek Sears, Christopher Brennan, Barbara Poulsen Nautrup, Jenifer Sheehy, Kristine Smith, Christopher Adolph

**Affiliations:** 1 Outcomes Research, Zoetis, Parsippany, NJ USA; 2 COVETRUS, Portland, ME USA; 3 Market Research, Zoetis, Parsippany, NJ USA; 4 EAH-Consulting, Aachen, Germany; 5 Veterinary Professional Services, Zoetis, Parsippany, NJ USA

**Keywords:** Heartworm, Flea, Tick, Purchase compliance, Retrospective analysis, Dog

## Abstract

**Background:**

Compliance failure with administration of heartworm (HW) disease preventives has been reported as the main contributor to HW disease incidence in medicalized dogs. This study aimed to evaluate purchase compliance with different canine HW preventive products in the USA.

**Methods:**

Anonymized transaction data from clinics throughout the USA served as the basis for two retrospective analyses. We first examined the monthly equivalent doses of HW preventive purchases from clinics that had implemented extended-release moxidectin injectables ProHeart^®^ 6 (PH6) and/or ProHeart^®^ 12 (PH12) compared to clinics that prescribed monthly HW preventatives (MHWP) only. In the second analysis, the purchase compliance in practices that dispensed only flea and tick (FT) and HW products separately but did not dispense combination products (dual-therapy practices) was compared to the purchase compliance with the combination product Simparica Trio^®^ (sarolaner, moxidectin, and pyrantel chewable tablets), purchased in clinics having implemented combination therapy in their formulary (combination-therapy practices). In both analyses, the numbers of monthly doses dispensed annually per dog were calculated.

**Results:**

Transaction data from 3,539,990 dogs in 4615 practices were included in the first analysis. In dogs administered PH12 or PH6, the numbers of monthly equivalent doses were 12 and 8.1, respectively. In both clinic types, the average annual number of MHWP doses totaled 7.3. In the second analysis, a total of 919 practices were identified as combination-therapy practices and 434 as dual-therapy-only practices. A total of 246,654 dogs (160,854 dogs in dual-therapy practices and 85,800 dogs in combination-therapy practices) were included in the calculation of the average annual number of monthly doses, which totaled 6.8 (HW preventive products) and 4.4 (FT products) in dual-therapy practices compared to 7.2 months for both FT and HW preventives with Simparica Trio^®^ across both practice types.

**Conclusions:**

The injectable HW preventive PH12 is the only product that provides 12 months of heartworm disease prevention in a single veterinarian-administered injection. When choosing a monthly preventive, the combination therapy was associated with a greater purchase compliance compared with FT and HW products being dispensed separately.

**Graphical Abstract:**

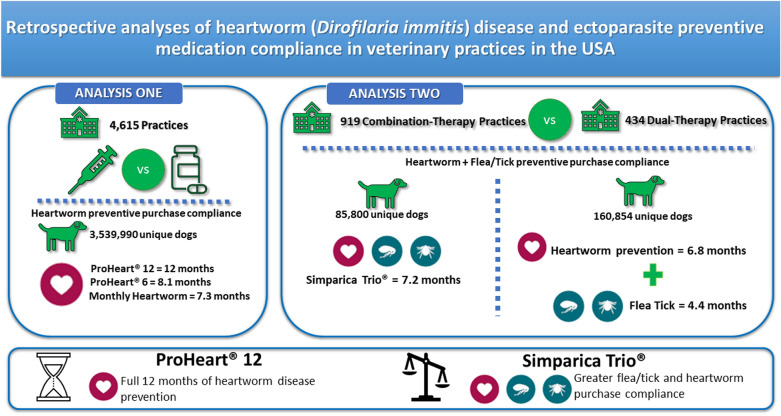

## Background

Canine heartworm (HW) disease is caused by the parasitic nematode *Dirofilaria immitis*, which has been reported in all states of the USA. Transmission occurs when competent mosquito vectors ingest *D. immitis* microfilariae from an infected host and then transmit infective third-stage larvae (L3) when feeding on a susceptible dog. The larvae mature and migrate within the tissues of the recipient dog, eventually reaching the pulmonary arteries and the heart, where even low worm burdens can produce life-threatening vascular pathology, especially in smaller breed dogs [1]. Despite improvements in diagnostic capability and increased availability of preventive products, HW disease is becoming more prevalent. A 2016 American Heartworm Society (AHS) survey reported a 21.7% increase between 2013 and 2016 in the average number of dogs diagnosed positive for adult HW per clinic [2]. In order to prevent HW disease, the AHS guidelines state that dogs should be on approved HW preventives year-round [1]. Compliance is a primary focus in HW prevention since it has been shown that most HW-positive dogs had received no HW prevention or experienced a gap in HW protection which provided an opportunity for development of the disease. The preventive approach for HW disease in dogs relies on a single drug class, macrocyclic lactones [1]. Resistance to macrocyclic lactones in *D. immitis* has been reported in the USA, primarily concentrated in the Mississippi delta [3], raising concerns of a potential loss of efficacy of this drug class. Yet most cases of suspected lack of effectiveness of HW preventives can still be explained by compliance rather than product failures [4].

In the USA, macrocyclic lactones can be administered in dogs by three routes: monthly oral (ivermectin, milbemycin oxime, moxidectin), monthly topical (moxidectin, selamectin), and parenteral (extended-release moxidectin injectable) [1, 5].

The extended-release moxidectin injectables ProHeart^®^ 6 (PH6, Zoetis Inc., Parsippany, NJ, USA) and ProHeart^®^ 12 (PH12, Zoetis Inc.) were specifically developed to help veterinarians address challenges with owner compliance in canine HW disease prevention [6], as a single dose protects dogs for 6 months [7] or 12 months [8], respectively. Drugs for monthly administration contain the macrocyclic lactone either as monotherapy or in combination with other active ingredients, to include efficacy against endo- and ectoparasites. The product Simparica Trio^®^ combines sarolaner, moxidectin, and pyrantel in a monthly chewable tablet (hereafter referred to as combination therapy) and was developed by the same manufacturer as PH6 and PH12 (Zoetis Inc.). It was the only combination product available in the USA at the time of data recording that provided combined protection from HW disease and most relevant fleas and ticks (FT), roundworms, and hookworms in dogs [5]. Similar to recommendations for HW disease prevention, the Companion Animal Parasite Council recommends FT preventive medication administration in dogs year-round and throughout their life [9, 10]. A recent retrospective analysis of transaction records in the USA, however, showed that 43% of dog owners purchased just one dose over the 12-month observation period [11].

The aims of this study were to use US transaction data to (1) evaluate the purchase compliance with the extended-release moxidectin formulations PH6 and PH12 compared to monthly HW preventives (MHWP) and (2) examine the purchase compliance with combination therapy compared to dual therapy with HW preventives and FT products.

## Methods

Two retrospective analyses were conducted, both using transaction data from a centralized database of more than 6000 practices across all states of the USA managed by Vetstreet Veterinary Practice Management Services (Covetrus, Inc., Portland, ME, USA). Covetrus provided anonymized transaction data from practices that met the inclusion criteria as defined for each of the two analyses.

Both analyses followed the guidelines and checklist for a systematic approach to compliance and persistence studies using retrospective databases [12].

### Retrospective analysis 1

Of all clinics in the database, practices were included in the analysis if they had records of HW preventive transactions for a full 12 months of the year. This excluded practices that were not actively recommending year-round HW prevention. All HW preventives that had to be given monthly (orally or topically) were grouped together as MHWP, being considered one preventive modality regardless of availability as a stand-alone or part of a combination product. The annually (PH12) or biannually (PH6) administered injectable moxidectin formulations were considered separately. Practices were classified as PH practices or non-PH practices. PH practices had to utilize PH6 and/or PH12 in any amount for at least 6 months during the year; otherwise, they were considered non-PH practices.

The observation period was from September 2019 until August 2020. Analyses were conducted at the patient level, considering only dogs that had a transaction for an HW preventive product. Canine patients without a documented purchase of HW preventives from the clinic were not included, as the aim was to examine purchase compliance with different HW preventives rather than the overall compliance with HW prophylaxis in the total medicalized dog population. Dogs with mixed product purchases such as MHWP plus PH6 or PH12 within the year’s observation period were excluded for the sake of clarity.

Transaction data were used to determine the number of purchased doses of MHWP, as well as the monthly equivalent doses of PH12 and PH6, with one injection of PH6 or PH12 corresponding to 6 or 12 monthly dose equivalents, respectively. Patients were grouped according to the length of the period of protection provided by the amount of MHWP dispensed over the 12-month observation period: 1–3 months, 4–5 months, 6 months, 7–9 months, 10–11 months, and 12 months, corresponding to an average of 2, 4.5, 6, 8, 10.5, and 12 monthly doses, respectively. The average annual doses per dog were calculated for MHWP, PH6, and PH12 as a weighted mean, considering the number of dogs assigned to the different time periods of protection, and all results were recorded separately for PH practices and non-PH practices.

### Retrospective analysis 2

In this analysis, two practice types were compared: practices that carried the combination product Simparica Trio^®^ (combination-therapy practices) and practices that dispensed FT products and HW preventives separately and did not use any combination product (dual-therapy practices). Combination-therapy practices had to dispense the combination product consistently following implementation in their formulary after the product was released on the US market in April 2020, with a minimum of 350 doses dispensed on average per month. The dual-therapy practices had to dispense FT-HW products consistently over the entire observation period (15 months), with an average minimum of 100 monthly FT-HW doses. The thresholds were chosen to ensure that only practices with significant HW medication sales were included in the analysis. Dual-therapy practices had no transaction data for any FT-HW combination product.

The observation period was from April 2020 to June 2021. In both practice types, dogs were identified that had a purchase documented for Simparica Trio^®^ (combination-therapy practices) or an FT and/or HW preventative (dual-therapy practices) between April 2020 and July 2020. These dogs were followed over the next 12 months. In dual-therapy practices, products were—*per definition*—prescribed separately and not necessarily on the same day. Depending on the products being purchased on the same day (FT-HW pair) or dispensed on separate days, different purchasing patterns could be observed (e.g., FT or HW only, or FT-HW pair plus FT only). All dogs with the same purchasing pattern were grouped together (purchasing pattern cohorts).

The following transactions led to exclusion of dogs in dual-therapy practices: (1) purchasing a combination product AND an FT or HW product on the same day, or (2) purchasing two drugs of the same product type (e.g., FT + FT) on the same day. Sentinel^®^ (milbemycin oxime + lufenuron) or Sentinel^®^ Spectrum^®^ (milbemycin oxime + lufenuron + praziquantel) were considered HW products (with lufenuron being an insect growth regulator rather than a flea adulticide). The use of nitenpyram (Capstar^®^) was not classified as flea prophylactic as it was licensed as infestation treatment only.

To allow for the calculation of the annual number of monthly doses of FT and HW preventives per dog, all dogs were grouped according to their purchasing pattern (dual-therapy practices) or defined as combination-therapy dogs. Annual doses per dog were calculated as a weighted mean, considering the number of dogs in each purchasing pattern cohort and presented separately for each cohort as well as for all dogs per practice type.

Additionally, revenues generated in dogs considered for the calculation of the number of purchased doses annually were analyzed. Revenues were recorded and combined for all dogs within a purchasing pattern cohort, considering revenues from FT and HW products as well as revenues from additional services. Revenues were then expressed per pet, considering the number of pets in each cohort, and total revenue per dog for all dogs in the two practice groups.

## Results

### Retrospective analysis 1

A total of 4615 practices met the inclusion criteria, i.e., were actively recommending HW preventive products. A total of 3,539,990 dogs were included in the calculation of the average monthly doses purchased annually per dog, from which 2,901,302 dogs were dispensed MHWP (1,430,032 in PH practices and 1,471,270 in non-PH practices) and 314,687 and 324,001 dogs received PH6 or PH12, respectively.

When considering MHWP only, in PH practices, most dogs were dispensed doses for 12 months, 6 months, or 2 months (34.2%, 29.6%, or 23.6%, respectively). The average annual number of doses per patient was 7.3. The same average annual number of MHWP doses per dog was calculated in non-PH practices, in which most dogs were dispensed doses for 6 months, 12 months, or 2 months of coverage (34.0%, 33.5%, or 22.0%, respectively). In PH practices, the use of PH 6 resulted in mean coverage for 8.1 months, as not all dogs received two injections within the 12-month observation period. The injection of PH12 resulted in 12 months of coverage in all treated dogs.

Overviews of the doses purchased annually per dog are presented in Table 1 (PH practices) and Table 2 (non-PH practices).

**Table 1:** Retrospective analysis 1: Results for practices that had implemented the extended-release moxidectin injectable ProHeart^®^ 6 (PH6) and/or ProHeart^®^ 12 (PH12) in their pharmacy stock

Product	Time period covered by doses dispensed annually	Corresponding monthly doses per year	Number of dogs	Proportion (%)	Monthly doses per dog dispensed annually
MHWP	1–3 months	2	338,067	23.6	
	4–5 months	4.5	57,150	4.0	
	6 months	6	423,880	29.6	
	7–9 months	8	91,655	6.4	
	10–11 months	10.5	30,433	2.1	
	12+ months	12	488,847	34.2	
	SUM		1,430,032	100.0	7.3
PH6	6 months	6	202,864	64	
	12 months	12	111,823	36	
	SUM		314,687	100	8.1
PH12	12 months	12	324,001	100	12.0

Dogs were classified according to the number of monthly doses (monthly heartworm preventives [MHWP]) or monthly equivalent doses (PH 6 or PH 12) that were dispensed over the 12-month observation period. Weighted average doses per dog were calculated, considering the number of dogs in each dose group

**Table 2:** Retrospective analysis 1: Results for practices that did not use the extended-release moxidectin injectable ProHeart^®^ 6 or ProHeart^®^ 12

Product	Time period covered by doses dispensed annually	Corresponding monthly doses per year	Number of dogs	Proportion (%)	Monthly doses per dog dispensed annually
MHWP	1–3 months	2	324,185	22.0	
	4–5 months	4.5	28,729	2.0	
	6 months	6	500,686	34.0	
	7–9 months	8	95,987	6.5	
	10–11 months	10.5	28,089	1.9	
	12+ months	12	493,594	33.5	
	SUM		1,471,270	100.0	**7.3**

Dogs were classified according to the number of doses of monthly heartworm preventives (MHWP) that were dispensed over the 12-month observation period. The weighted average dose per dog was calculated, considering the number of dogs in each dose group

### Retrospective analysis 2

A total of 919 practices were identified as combination-therapy practices and 434 as dual-therapy practices. The number of dogs included in the calculation of the average number of monthly doses and revenues was 246,654 (160,854 dogs in dual-therapy practices and 85,800 dogs in combination-therapy practices).

In dual-therapy practices, the majority (over 85%) of pairings, i.e., the combination of two products dispensed on the same day, included an isoxazoline product, with afoxolaner + ivermectin being the most often purchased product pair. All other combinations of FT and HW products were more rarely dispensed simultaneously (Table 3). However, FT and HW preventive products could also be dispensed on separate days or in combination with paired products, resulting in different purchasing patterns, as presented in Table 4.

**Table 3:** Retrospective analysis 2: Overview of combinations dispensed on the same day in practices that sold flea and tick (FT) products and heartworm (HW) preventives separately, i.e., did not use combination products

Product pair (FT, HW)	Active ingredient (FT, HW)	Percentage (%)
Nexgard® ^a^, Heartgard® ^a^	Afoxolaner, ivermectin	42
Credelio® ^a^, Interceptor/Plus® ^a^	Lotilaner, milbemycin oxime (with or without praziquantel)	11
Bravecto® ^a^, Interceptor/Plus® ^a^	Fluralaner, milbemycin oxime (with or without praziquantel)	11
Bravecto® ^a^, Heartgard® ^a^	Fluralaner, ivermectin	8
Nexgard® ^a^, Interceptor/Plus® ^a^	Afoxolaner, milbemycin oxime (with or without praziquantel)	4
Frontline/Gold® ^b^, Heartgard® ^a^	Fipronil (with or without methoprene and pyriproxyfen), ivermectin	3
Bravecto® ^a^, Proheart® 6 ^c^	Fluralaner, moxidectin	3
Bravecto® ^a^, Tri-Heart® ^a^	Fluralaner, ivermectin	3
Simparica® ^a^, Interceptor/Plus® ^a^	Sarolaner, milbemycin oxime (with or without praziquantel)	2
Bravecto® ^a^, Proheart® 12 ^c^	Fluralaner, moxidectin	2
Simparica® ^a^, Heartgard® ^a^	Sarolaner, ivermectin	1
Nexgard® ^a^, Proheart® 12 ^c^	Afoxolaner, moxidectin	1
All others		10

Application: ^a^ oral; ^b^ topical; ^c^ injection

**Table 4:** Retrospective analysis 2: Overview of the purchasing patterns observed in practices that sold flea and tick (FT) products and heartworm (HW) preventives separately, i.e., did not use combination products

Purchasing pattern (abbreviation)	Purchasing pattern (definition)
FT only	The dogs received FT medication at any time, but no HW preventative during the observation period
HW only	The dogs received HW preventive(s) at any time, but no FT medication during the observation period
FT only + HW only	The dogs received FT and HW preventives on different days during the observation period
FT-HW pair	The dogs received FT and HW preventives on the same day
FT-HW pair + FT only	The dogs received FT and HW preventives on the same day and additional FT medication on another day
FT-HW pair + HW only	The dogs received FT and HW preventives on the same day and additional HW preventive(s) on another day
FT-HW pair + FT only + HW only	The dogs received FT and HW medication on the same day and additional FT and HW preventives on different days

Each purchasing pattern was handled as a single cohort and results were recorded for each cohort separately

In dual-therapy practices, a total of 738,733 and 1,086,656 monthly doses of FT and HW products were dispensed, respectively. An overview of the monthly doses purchased for each purchasing pattern cohort is presented in Table 5. The calculated annual doses per dog for FT products varied between 0.0 (only HW preventatives purchased) and 10.1 (in dogs that were dispensed FT-HW simultaneously and FT products separately). The corresponding annual doses per dog for HW preventives varied between 0.0 (only FT products purchased) and 12.7 (in dogs that were dispensed FT-HW products simultaneously as well as separately). Over all dogs and purchase pattern cohorts, the average monthly doses were calculated as 4.4 for FT products and 6.8 for HW preventives over the 12-month observation period. Combination-therapy practices dispensed 614,262 monthly doses of combination therapy for 85,800 patients, corresponding to 7.2 monthly doses for FT and HW products over 12 months (Table 5).

**Table 5:** Retrospective analysis 2: Summary of monthly doses of flea and tick (FT) and heartworm (HW) preventive products dispensed over the 12-month observation period

Purchasing pattern cohorts	*Patients*	Number of monthly doses dispensed over the 12-month observation period				
		FT doses	HW doses	FT + HW doses total	FT doses per dog	HW doses per dog
**Dual-therapy practices**						
FT only	*44,666*	237,362	0	237,362	5.3	0.0
HW only	*49,890*	0	466,257	466,257	0.0	9.3
FT only + HW only	*13,113*	84,613	121,049	205,662	6.5	9.2
FT-HW pair	*29,328*	206,003	249,126	455,129	7.0	8.5
FT-HW pair + FT only	*11,214*	113,251	94,943	208,194	10.1	8.5
FT-HW pair + HW only	*6745*	37,704	80,363	118,067	5.6	11.9
FT-HW pair + FT only + HW only	*5898*	59,800	74,918	134,718	10.1	12.7
Total	160,854	738,733	1,086,656	1,825,389	4.4	6.8

Purchasing pattern cohorts	*Patients*	Number of monthly doses dispensed over the 12-month observation period				
		FT doses	HW doses	FT + HW doses total	FT doses per dog	HW doses per dog
**Combination-therapy practices**						
Combination-therapy TOTAL	85,800	614,262	614,262	614,262	7.2	7.2

Results were recorded separately for the purchase pattern cohorts as defined in Table 4 for dual-therapy practices (practices that sold FT products and HW preventives separately, i.e., did not use combination products) and for Simparica Trio^®^ (combination therapy). Annual doses per dog were calculated for each purchase pattern cohort and weighted average doses were calculated for dual-therapy and combination-therapy practices, considering the number of dogs in each purchasing pattern cohort

The total revenue achieved in dual-therapy practices for the 160,854 dogs was $47,541,809, nearly equally distributed to FT + HW products (49.2%) and other additional services (50.8%). The average total revenue per dog was calculated as $295.56 over 12 months. In combination-therapy practices, a slightly higher percentage of total revenues was generated in the 85,800 dogs with additional services ($17,802,845; 52.3%) compared to revenues from FT + HW products ($16,230,713; 47.7%). Total revenues per dog were calculated as $396.66 annually (Table 6).

**Table 6:** Retrospective analysis 2: Summary of the revenues generated over the 12-month observation period for dogs included in the analyses of monthly doses

Purchase pattern cohorts	*Patients*	Revenues generated over the 12-month observation period					
		Total FT-HW revenue	Total additional revenue	Total revenue	FT-HW revenue per dog	Additional revenue per dog	Average total revenue per dog
**Dual-therapy practices**							
FT only	*44,666*	$4,372,354	$3,729,598	$8,101,952	$97.89	$83.50	$181.39
HW only	*49,890*	$3,943,702	$7,576,637	$11,520,338	$79.05	$151.87	$230.91
FT only + HW only	*13,113*	$2,559,390	$2,567,675	$5,127,065	$195.18	$195.81	$390.99
FT-HW pair	*29,328*	$6,291,415	$4,963,557	$11,254,972	$214.52	$169.24	$383.76
FT-HW pair + FT only	*11,214*	$3,015,912	$2,328,455	$5,344,367	$268.94	$207.64	$476.58
FT-HW pair + HW only	*6745*	$1,435,107	$1,537,452	$2,972,559	$212.77	$227.94	$440.71
FT-HW pair + FT only + HW only	*5898*	$1,788,943	$1,431,613	$3,220,556	$303.31	$242.73	$546.04
Total	160,854	$23,406,823	$24,134,987	$47,541,809	$145.52	$150.04	$295.56

Purchase pattern cohorts	*Patients*	Revenues generated over the 12-month observation period					
		Total FT-HW revenue	Total additional revenue	Total revenue	FT-HW revenue per dog	Additional revenue per dog	Average total revenue per dog
**Combination-therapy practices**							
Combination therapy TOTAL	85,800	$16,230,713	$17,802,845	$34,033,558	$189.17	$207.49	$396.66

Revenues were recorded separately for flea and tick (FT) and heartworm (HW) preventive products as well as additional services. Results were reported for each purchasing pattern cohort as defined in Table 4 for dual-therapy practices (i.e., practices that sold FT products and HW preventatives separately, i.e., did not use combination products) and Simparica Trio^®^ (combination therapy). Weighted annual revenues per dog were calculated for dual-therapy and combination-therapy practices, considering the number of dogs in each purchasing pattern cohort

## Discussion

We used transaction data from veterinary clinics across the USA to examine dog owner purchase compliance with HW preventive drugs. Several limitations need to be considered when interpreting transaction data. First, the purchase history of a medication is an imperfect substitute for the number of doses of medication that a pet successfully receives; it only reflects the maximum doses that a pet owner purchased in the clinic [13]. This limitation applies to oral or topical HW preventives only, as the injectables PH6 and PH12 put compliance in the veterinarian’s control. Second, transaction data from veterinary clinics cannot capture purchases made outside the veterinary clinic, i.e., do not account for potential prescriptions of MHWP filled through online pharmacies, thereby potentially leading to an underestimation of the actual purchase compliance. The third limitation is the possibility of an overestimation of compliance if purchases are prescribed to the individual patient in the respective clinic but will be divided up among other dogs from the same household. This fact has been reported as a reason for the incidence of HW disease in dogs presumed to be 100% compliant [4]. The second and third limitations do not apply to injectable HW prevention that is restricted to the use by or on the order of a licensed veterinarian [7, 8], but cannot be ruled out for MHWP. Despite this, retrospective analyses of transaction data have been used previously to examine purchase compliance with antiparasitics in dogs [6, 11, 13–16].

In our first analysis, the calculated number of average annual purchases of MHWP was 7.3 per dog, calculated for PH practices and non-PH practices. These dogs were unprotected for more than 4 months of the year against HW disease, and at risk of developing a potentially fatal disease once exposed during the coverage gap. As reservoirs of HW microfilariae, HW-positive dogs put other dogs at risk and risk the spread of macrocyclic lactone-resistant isolates of *D. immitis*.

The injectable moxidectin PH6 offers half a year of protection, corresponding to six doses of MHWP per injection. Considering the number of injections a single dog received over the 12-month observation period, the average monthly equivalent was 8.1, better than the average annual dose of MHWP but still less than optimum. The injectable moxidectin PH12 offers a full year of protection with one injection corresponding to 12 monthly equivalent doses in all treated dogs. It should be noted that PH12 not only provides 100% compliance over 12 months but has also been shown to be 100% effective in a large field trial, whereas in the positive control arm 4 of 218 dogs that received ivermectin + pyrantel (Heartgard Plus^®^) tested positive for HW during the 20-month study period. All treatment failures with ivermectin occurred in the Lower Mississippi River Valley, where confirmed cases of macrocyclic lactone resistance have been primarily concentrated. In this field trial, compliance was rigorously documented, and the authors assessed the lack of preventive effectiveness in the ivermectin group as unlikely to be due to compliance failure under the artificial study conditions [17]. In view of the existence of macrocyclic lactone-resistant isolates of *D. immitis*, the prevention of HW disease is best achieved using optimized formulations of HW preventives regarding compliance and efficacy.

We did not include an economic calculation in the first analysis, as the revenue benefits of implementing PH6 and PH12 as a strategy to prevent HW disease have been demonstrated previously in two retrospective analyses of transaction data [6, 16]. A pharmacoeconomic analysis evaluated the impact of incorporating PH6 into the practice formulary. Although PH6 was similarly priced to the owner as six doses of MHWP, 85% of patients on injectable moxidectin recorded additional transactions during the first visit (average invoice $161) compared with only 55% of pet owners who purchased MHWP (average invoice $141) [16]. In the other economic study, the implementation of PH12 resulted in 15% growth in preventive revenue compared to 3.9% growth in practices that did not bring on PH12, although the cost of PH12 was equivalent to 12 doses of MHWP [6].

In our second retrospective analysis, we compared the purchase compliance for the FT-HW combination product with purchases of single FT and HW preventive products. Combination-therapy patients were dispensed on average 7.2 monthly doses annually (FT and HW compliance), compared to 6.8 monthly doses of HW and 4.4 monthly doses of FT preventives when dispensed separately.

We cannot exclude a potential underestimation of compliance due to purchases outside the veterinary clinic. It can be rationally assumed that this limitation applies to both groups, as all products of interest were subject to administration by the dog owner. Similarly, we cannot rule out a potential overestimation of compliance if doses were shared among different dogs in multi-pet households. In dual-therapy practices, some purchasing patterns included numerous purchases of different products at different time points (e.g., FT-HW pair + FT only + HW only) and might be indicative of multiple dog purchases.

Although not the primary aim of our analysis, we compared the revenues registered in dual-therapy and combination-therapy practices. Combination therapy resulted in approximately $50 additional revenue generated through additional services per dog compared with dogs in dual-therapy practices. The inclusion criteria between the two practice groups differed, as the number of monthly dispensed doses required for inclusion was higher in the case of combination-therapy practices. The high number of doses that combination-therapy clinics had to dispense for inclusion was chosen to exclude practices that had implemented combination therapy shortly after launch but did not stock the product afterwards. It is possible that the combination-therapy and dual-therapy practices differed in terms of prescribing patterns and clientele, thereby affecting additional services offered by the veterinarians, and results of additional revenues generated should be interpreted with caution. Selection criteria were based on sales volume and not geographical distribution of the two practice groups. Because year-round HW prophylaxis is recommended throughout the USA [1], this likely reduces the impact of differences in the geographical distribution of practices on the results of HW purchase compliance. However, we cannot exclude the possibility that any potential differences in regional clinic distribution might have influenced our results for FT preventives compliance and revenues generated with FT products or additional services.

There are aspects involving both retrospective analyses that deserve consideration. Both analyses' inclusion criteria ensured that clinics were actively recommending HW preventive products, i.e., dispensing them year-round. It can be assumed that annual doses per dog dispensed in other clinics that are less active in recommending HW preventives are even lower. Our results do not reflect the annual compliance in the overall canine population. We did not differentiate purchase compliance between oral and topical products for monthly HW prevention, as both administration forms were grouped together in both analyses. It has been reported that improper owner administration of topical products can lead to inadequate exposure and absorption of the active ingredients, and subsequently to sub-efficacious doses to the dog [3]. This risk of potential underdosing with topical HW products was not captured in our analyses.

Concerning the validity of our study, estimates of average doses of MHWP purchased annually per dog were consistent throughout our analyses, resulting in similar annual doses of MHWP in PH practices (7.3), non-PH practices (7.3), and combination-therapy practices (7.2). Only dual-therapy practices had somewhat lower average annual doses of MHWP (6.8). The same data source was used for the two retrospective analyses. Different inclusion criteria resulted in different patients being considered in the two analyses, but the results are comparable. Our combination-therapy estimate is also comparable to the average of 7.13 annual doses per dog calculated for monthly FT products in another retrospective analysis of transaction data using a different data source and different inclusion criteria [13].

## Conclusions

The injectable HW preventive PH12 is the only product that inherently provides 12 months of HW disease prevention in a single veterinarian-administered injection. When choosing a monthly preventive, the combination therapy is associated with greater purchase compliance than FT and HW products being dispensed separately, and also provides broad protection against multiple endo- and ectoparasites.

## Data Availability

Data supporting the conclusions of this article are included within the article.
